# Determinants of depressive symptom trajectories in self-reported chronic obstructive pulmonary disease patients

**DOI:** 10.1186/s12890-022-02060-5

**Published:** 2022-07-17

**Authors:** Cui Wang, Hongbo Chen, Peiyuan Liu, Ziqiu Zou, Shaomei Shang

**Affiliations:** 1 Peking University School of Nursing, 38 Xueyuan Road, Haidian District, Beijing, China; 2 Peking University School of Public Health, Beijing, China

**Keywords:** COPD, Depressive symptoms, Trajectory pattern, Latent class growth modeling

## Abstract

**Background:**

The depressive symptom trajectories of COPD individuals and its’ predictors remain to be established. Therefore, this study aimed to explore the trajectories of depressive symptoms and predictors thereof in COPD patients.

**Methods:**

A total of 1286 individuals over 45 years of age with self-reported COPD were assessed. Depressive symptoms were evaluated using the Center for Epidemiological Studies Depression Scale short form, with depressive symptom trajectories being identified via latent class growth analysis. The predictors of depressive symptom trajectories were then identified through multinomial logistic regression.

**Results:**

Finally, three depressive symptom trajectories were identified: “steadily high”, “consistently moderate”, and “consistently low”. Old age, longer night-time sleep duration, and high BMI were found to be associated with individuals being classified under the “consistently moderate” trajectory. Moreover, participants exhibiting more than two chronic conditions were more likely to be classified under the “consistently moderate” trajectory. Higher education and lower hand grip strength were important predictors of individuals classified in the “steadily high” trajectory.

**Conclusions:**

To conclude, three depressive symptom trajectories were identified in self-reported COPD individuals. To ensure timely intervention aimed at preventing the worsening of depressive symptom progression among COPD individuals, health-care workers should regular analyze depressive symptoms and provide appropriate interventions when possible.

## Introduction

Chronic obstructive pulmonary disease (COPD) is a major threat to global public health that is characterized by progressive airflow limitations that are not fully reversible [1]. Approximately 174.5 million adults in the world were estimated to suffer from COPD as of 2015 [2, 3], including approximately 99.9 million adults in China [4], where COPD was identified as the third most prominent cause of mortality [5]. Further research is thus essential to characterize COPD-related morbidity and mortality in China.

COPD patients commonly exhibit some level of depressive symptoms, with depressive symptoms being in 16–74% of them, and the symptoms are commonly unrecognized and thus go untreated [6]. The unrecognized depressive symptoms are a major clinical challenge as they can adversely affect physical health, quality of life, and prognosis of COPD individuals [7, 8]. Depressive symptoms are complex and often change dynamically with the passage of time, with many patients exhibiting few or no depressive symptoms consistently, whereas a minority report persistent symptoms [9]. Previous systematic review have identified either three or four depressive symptoms trajectories that vary according to their stability and severity, further supporting the heterogeneity of depressive symptoms [10]. Gaining insight regarding depressive symptom trajectories has important implications for current understanding of the burden of depression among the particular patient populations, potentially guiding preventative treatments and other appropriate interventions. In previous reports, depressive symptom trajectories have been characterized in older adults [11, 12], breast cancer patients [13], and diabetes patients [14]. However, few such studies have been performed in COPD population.

In addition to assessing changes in intrapersonal depressive symptoms with the passage of time, prior reports have also sought to identify factors correlated with these different trajectories that differ among individuals [11, 15]. Factors including sex, age, and physical functioning have all previously been found to influence depressive symptom trajectories [11]. Other study has reported these trajectories to be associated with functional impairment status [16]. In COPD individuals, cross-sectional analyses have also sought to characterize the determinants of depressive symptoms [17, 18]. However, these studies primarily evaluated these variables at a particular time point. Identifying baseline predictors of depressive symptom trajectories, in contrast, has the potential to guide more effective and timely interventional efforts that have a greater chance of improving health-related outcomes of COPD patients. At present, the predictors of COPD patient depressive symptom trajectories, however, remain to be established.

### Purpose

This study aimed to evaluate depressive symptom trajectories and the baseline determinants thereof in self-reported COPD patients.

## Methods

### Participants

Data were collected from the China Health and Retirement Longitudinal Study (CHARLS), which is a national database that compiles longitudinal survey data pertaining to a population of older adults ≥ 45 years of age. Since beginning in 2011, this survey has covered 450 village-level units of 150 counties and 28 provinces in China [19], with regular follow-up using the same biomedical instruments and questionnaires being conducted every 2 years [19]. The survey was not conducted during an outpatient or hospital stay. Eligible survey subjects were selected using a four-stage, stratified, cluster probability sampling strategy [19], and all provided written informed consent to participate. In addition, ethical approval for all the CHARLS waves were obtained from the Institutional Review Board (IRB) at Peking University (IRB approval number: IRB00001052-11015).

Properly trained research assistants conducted in-person interviews with eligible participants to collect information including sociodemographic details, health-related information, and clinical measures [19]. The COPD diagnoses in the CHARLS survey were previously published [1]. Specifically, study subjects were asked whether or not a physician had diagnosed them with COPD, with a response of “yes” being used to define participants with COPD.

The CHARLS survey data have been released in four waves. However, the wave-4 only released partial data, excluding anthropometric measurements. Furthermore, as wave-4 was performed in 2018, we only utilized data from wave-1 (2011; 17,708 respondents), wave-2 (2013; 18,612 respondents), and wave-3 (2015; 21,095 respondents) to ensure a consistent time interval in our longitudinal analysis. Participants were excluded if: (1) they did not have COPD (n = 19,314); or (2) they were under 45 years of age (n = 19). In total, 1762 participants with COPD that were 45 years of age or older completed baseline questionnaires pertaining to depressive symptoms, of whom 1120, 1070, and 1286 completed the first follow-up survey, the second follow-up survey, and either the first or second follow-up survey, respectively. Finally, the current analysis was conducted among the 1286 participants.

### Measurements

#### Dependent variables

Depressive symptoms were analyzed using the Center for Epidemiological Studies Depression Scale (CES-D) short form, which consists of 10 items scored from 0 (rarely/none of the time) to 3 (most/all of the time) with a Likert scale. Total scores ranged from 0 to 30, with items 5 (‘feeling hopeful about the future’) and 8 (‘feeling happy’) being scored in reverse. Higher total CES-D scores were consistent with increased depressive symptoms, and a score of 12 or higher has been used as the cut-off point for depressive symptoms [20].

#### Independent variables

The independent variables were included according to studies related to influencing factors of depressive symptoms of COPD patients [17, 18]. In addition, our authors discuss the inclusion of variables. Overall, the independent variables were screened for both clinical and statistical significance.

Sociodemographic and health-related data were collected in this study. Sociodemographic variables included sex, age, marital status (married/living together or other [including widowed, divorced, or unmarried]), education level (elementary school or below, middle school, high school or above), and residence setting (rural or urban).

Health-related variables consisted of self-reported variables and anthropometric measures. Self-reported information included smoking status (current, former, or never), sleep duration (answered in response to the question: “During the past month, how many hours of actual sleep did you get at night [average hours for one night]?”), number of chronic conditions (based on the physician-diagnosed presence or absence of cancer, diabetes, stroke, hypertension, dyslipidemia, liver disease, heart problems, kidney disease, gastrointestinal disease, psychiatric problems, asthma, memory-related conditions, arthritis or rheumatism), and self-rated health (SRH) in response to the question “Would you say your health is excellent, very good, good, fair, or poor?”, with patients responding “excellent”, “very good”, or “good” being classified as ‘good’, and those responding “fair” or “poor” being classified as poor. The basic activities of daily living (BADL) (dressing, bathing, eating, getting into or out of bed, using the toilet, and continence control) and instrumental activities of daily living (IADL) tasks (cooking, cleaning, shopping, managing assets, and taking medications) were used to evaluate the disability status of each patient. For each of these tasks, participants were asked, “Do you have difficulty in performing the task?” A BADL or IADL disability was considered to be present for participants responding with the following statements to one or more tasks: “I have difficulty but can still do it,” “Yes, I have difficulty and need help,” or “I cannot do it”.

Anthropometric measurements included peak expiratory flow (PEF), body mass index (BMI), hand grip strength (HGS), pulse, systolic pressure (SBP), and diastolic pressure (DBP). PEF (L/min) was measured based on the average of three readings collected using a peak flow meter (Shanghai, China). BMI (kg/m^2^) was determined by dividing body weight by height squared for each patient. HGS (kg) was approximated with a dynamometer (WL-100, Nantong, China). While standing with their hand naturally at their sides, participants were directed to squeeze the handles as hard as they were able, with the maximum measure from two tests of the dominant hand being recorded. Blood pressure was assessed three times (approximately 45 s apart) for each participant on the left arm in a sitting position using an electronic blood pressure monitor (Omron HEM-7112). The recorded value was the average of three measurements.

### Statistical analysis

SPSS version 22.0 (IBM Corp, NY, 2012) and Mplus (version 8.0; Muthén & Muthén, Los Angeles, CA) were used for all statistical testing. Latent class growth analysis (LCGA), which is a longitudinal technique derived from conventional growth modeling, was employed to analyze and identify distinct depressive trait trajectories. While conventional analyses assumed that all individuals within a particular study group are derived from a single population, for which one average trajectory can be used to adequately describe changes in a given pattern for that population [21], the LCGA relax this assumption such that individuals can be members of multiple latent underlying sub-populations [21]. LCGA primarily seeks to characterize the numbers and characteristics of these populations via the identification of the *k* number of distinct latent classes (i.e., subgroups), which were depressive symptom trajectories in the current study. Each of these identified classes exhibits particular growth parameters (slope, intercept) that are assumed to be latent [21].

Optimal numbers of latent classes were determined based upon Akaike Information Criterion (AIC), Bayesian Information Criterion (BIC), Bootstrapped likelihood ratio test (BLRT), entropy, and percent of participants per class values. AIC and BIC values were initially considered, with lower values being indicative of better model fit. For BIC, a reduction of a minimum of 10 points was considered indicative of sufficient improvement. BLRT values were then taken into consideration, with higher entropy being indicative of better fit. Optimal class number was determined by assessing the posterior probabilities for each individual in the sample (the average posterior probability of group assignment > 0.7), entropy (with values closer to 1 being more favorable), and clinical interpretability.

Baseline characteristics were performed with descriptive analyses. Continuous variables were given as means ± standard deviation (SD) and medians (interquartile range [IQR]) when normally and non-normally distributed, respectively, whereas categorical variables were given as frequencies (%). One-way ANOVAs were used to compare differences in CES-D scores among time points, with missing data being filled via multiple imputation using Bayesian methods [22]. Baseline predictors of different depressive symptom trajectories were identified via multinomial logistic analysis. A two-sided *P* < 0.05 was the threshold of statistical significance.

### Findings

#### Patient characteristics

The 1286 participants had an average age of 61.4 years, and 56.8% were female. The majority (80.1%) of participants reported living in rural areas, and 73.6% reported having an educational level of primary school or below. At baseline, the mean CES-D score for these subjects was 10.5, and the percentages of missing values ranged from 0.5 to 25.0% for different predictors in this analysis. Baseline patient characteristics and details regarding these missing values are compiled in Table 1.

Between 2011 and 2013, the mean CES-D score declined, while it rose in 2015, with respective average score at these time points of 10.5 (*SD* = 6.7), 9.6 (*SD* = 6.6), and 10.0 (*SD* = 7.0). There was a significant difference in the CES-D score from 2011 to 2015 (*F* = 5.076, *P* = 0.006).

**Table 1:** Baseline characteristics of participants in 2011

Variable	Distribution	Number of imputed data
Age (years), mean(SD)	61.4 (9.3)	0
Male, n (%)	731 (56.8)	0
Rural, n (%)	1030 (80.1)	10
Education level, n (%)		0
Elementary school or below	946 (73.6)	
Middle school	214 (16.6)	
High school or above	126 (9.8)	
Currently married, n (%)	1065 (82.8)	0
Smoking status, n (%)		0
Current smoker	610 (47.4)	
Former smoker	207 (16.1)	
None smoker	469 (36.5)	
Drinking status, n (%)		322
Current drinker	896 (69.7)	
Former drinker	193 (15.0)	
None drinker	197 (15.3)	
Sleeping duration (h), mean (SD)	5.9 (2.0)	8
Chronic conditions, n (%)		70
0–2	851 (66.2)	
> 2	435 (33.8)	
SRH, n (%)		0
Poor	1155 (89.8)	
Good	131 (10.2)	
CES-D score	10.5 (6.7)	0
Depressive symptoms		0
Yes	764 (59.4)	
No	522 (40.6)	
BADL disability, n (%)	353 (27.4)	212
IADL disability, n (%)	374 (29.1)	6
BMI (kg/m^2^), mean (SD)	22.5 (4.1)	176
HGS (kg), mean (SD)	30.1 (10.1)	193
Pulse (bpm), mean (SD)	74.4 (11.1)	172
Systolic pressure (mmHg), mean (SD)	131.5 (21.5)	170
Diastolic pressure (mmHg), mean (SD)	75.5 (12.2)	171
PEF (l/min), mean (SD)	209.6 (108.6)	216

*SRH* self-rated health, *CES-D scale* Center for Epidemiological Studies Depression scale, *BADL* basic activities of daily living, *IADL* instrumental activities of daily living, *BMI* body mass index, *HGS* hand grip strength, *PEF* peak expiratory flow

#### Identifying depressive symptom trajectory patterns

**Fig. 1:**
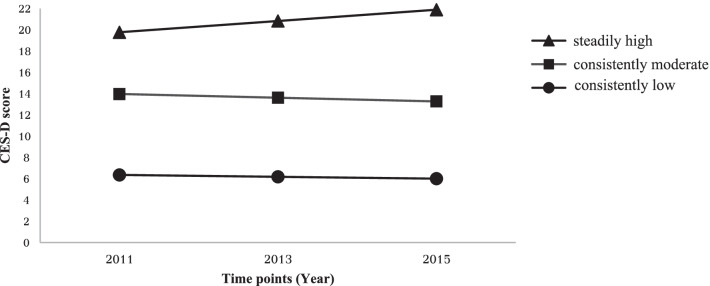
Three trajectories of CES-D scores across three time points

Classification fit statistics are showed in Table 2. When increasing from a one-class to a five-class model, AIC and BIC values declined. Furthermore, entropy was relatively higher in two-class and three-class model. The percentages of participants in each class in the two-class model were 70.8% and 29.2%, while in the three-class model these percentages were 8.4% (class 1), 33.7% (class 2), and 57.9% (class 3), respectively. While one of these classes incorporated a relatively small number of patients, we opted to utilize a three-class model based on goodness-of-fit indices recommended in prior research [23]. The developed four- and five-class models were not convergent. Based on the observed characteristics of these distributions, the three identified depressive symptom trajectories were defined as “steadily high” (n = 108, 8.4%), “consistently moderate” (n = 434, 33.7%), and “consistently low” (n = 744, 57.9%), with CES-D score-based trajectories being shown in Fig. 1. For participants classified under the “steadily high” trajectory, the CES-D scores continued to rise across the three analyzed time points, with these values being higher than those for the two other trajectories. The CES-D scores for participants classified under the “consistently moderate” trajectory remained at relatively stable intermediate levels, while for individuals classified under the “consistently low” trajectory, the CES-D scores tended to remain stable and were lower than those for the two other trajectories.

**Table 2:** Fit statistics of latent profile analysis

Model	Likelihood	AIC	BIC	Entropy	BLRT	Participants per class (%)
1	− 12802.6	25615.3	25641.1	–	–	–
2	− 12328.2	24672.3	24713.6	0.787	< 0.001	910 (70.8) 376 (29.2)
3	− 12239.0	24499.9	24556.7	0.748	< 0.001	108 (8.4) 434 (33.7) 744 (57.9)
4	− 12212.5	24452.9	24525.2	0.701	< 0.001	165 (12.8) 308 (24.0) 134 (10.4) 679 (52.8)
5	− 12197.0	24427.9	24515.6	0.702	0.043	625 (48.6) 42 (3.3) 134 (11.1) 320 (24.9) 156 (12.1)

*AIC* Akaike Information Criterion, *BIC* Bayesian Information Criterion, *BLRT* Bootstrapped likelihood ratio test

#### Determinants of depressive symptom trajectory patterns

Using the “consistently low” trajectory as a reference group, a multinomial logistic regression was conducted to identify determinants of depressive symptom trajectories. This analysis revealed that old age (*OR *1.017, 95%CI 1.001–1.034, *P =* 0.033) and longer duration of nighttime sleep (*OR* 1.077, 95%CI 1.011–1.046, *P =* 0.021) were associated with an increased risk of participants being classified under the “consistently moderate” trajectory (Table 3). Participants exhibiting more than two chronic conditions (*OR *1.383, 95%CI 1.058–1.809, *P =* 0.018) were more likely to be classified under the “consistently moderate” trajectory. High BMI (*OR *1.047, 95%CI 1.013–1.082, *P* = 0.007) was linked to participants being classified under the “consistently moderate” trajectory. In addition, compared with participants with elementary school or below, patients with middle school or high school were more likely to be classified under the “steadily high” trajectory (*OR* 2.000, 95%CI 1.057–3.782, *P* = 0.033; *OR *1.756, 95%CI 1.010–3.052, *P =* 0.046). Lower HGS (*OR * 0.972, 95%CI 0.947–0.996, *P =* 0.025) was associated with higher odds of being classified under the “steadily high” trajectory.

**Table 3:** Determinants of trajectories for depressive symptoms

Variable	Consistently moderate versus consistently low			Steadily high versus consistently low		
	*OR*	*95%CI*	*P*	*OR*	*95%CI*	*P*
Age	**1.017**	**1.001–1.034**	**0.033**	0.994	0.968–1.021	0.656
Sex						
Female		Reference			Reference	
Male	0.924	0.641–1.332	0.673	0.957	0.512–1.790	0.892
Rural	1.230	0.890–1.699	0.211	1.137	0.664–1.947	0.640
Education level						
Elementary school or below		Reference			Reference	
Middle school	0.796	0.507–1.251	0.323	**2.000**	**1.057–3.782**	**0.033**
High school	1.198	0.851–1.686	0.301	**1.756**	**1.010–3.052**	**0.046**
Marital status						
Others*		Reference			Reference	
Currently married	1.093	0.786–1.521	0.597	1.033	0.589–1.810	0.911
Smoking status						
Non smoker		Reference			Reference	
Current smoker	1.350	0.953–1.914	0.091	1.372	0.757–2.487	0.297
Former smoker	1.351	0.892–2.045	0.155	1.520	0.750–3.083	0.245
Drinking status						
None drinker		Reference			Reference	
Current drinker	0.781	0.547–1.113	0.171	0.544	0.272–1.087	0.085
Former drinker	0.715	0.495–1.033	0.074	0.874	0.471–1.622	0.670
Sleep duration	**1.077**	**1.011–1.146**	**0.021**	1.009	0.905–1.124	0.871
Chronic conditions						
0–2		Reference			Reference	
> 2	**1.383**	**1.058–1.809**	**0.018**	1.077	0.680–1.706	0.752
SRH						
Good		Reference			Reference	
Poor	0.879	0.586–1.319	0.535	0.596	0.325–1.094	0.095
BADL disability						
No		Reference			Reference	
Yes	0.763	0.560–1.039	0.086	0.760	0.439–1.315	0.326
IADL disability						
No		Reference			Reference	
Yes	1.089	0.803–1.477	0.582	0.844	0.489–1.455	0.541
BMI	**1.047**	**1.013–1.082**	**0.007**	0.997	0.945–1.053	0.919
HGS	1.005	0.990–1.021	0.495	**0.972**	**0.947–0.996**	**0.025**
Pulse	0.996	0.984–1.007	0.451	0.998	0.979–1.018	0.867
Systolic pressure	0.998	0.989–1.008	0.743	0.997	0.981–1.012	0.674
Diastolic pressure	1.001	0.985–1.018	0.878	1.015	0.987–1.044	0.287
PEF	1.001	0.999–1.002	0.310	0.999	0.997–1.002	0.616

Bold indicates statistical significance

Others*: including widowed, divorced, or unmarried; *SRH* self-rated health, *CES-D scale* Center for Epidemiological Studies Depression scale, *BADL* basic activities of daily living, *IADL* instrumental activities of daily living, *BMI* body mass index, *HGS* hand grip strength, *PEF* peak expiratory flow

## Discussion

To our knowledge, this is the first study to assess depressive symptom trajectories over a 4-year period among middle-aged and older self-reported COPD patients in China. Three distinct depressive symptom trajectories were identified in the current study. Over half of participants (57.9%) were classified under the “consistently low” trajectory in which CES-D scores were consistently lower than those for other trajectories at all three time points. This may be indicative of the relatively good quality of care provided to COPD patients in China, and suggests that a majority of patients are able to adapt to their disease status in a consistent manner. However, a large proportion (42.1%) of self-reported COPD patients exhibited relatively high CES-D scores, with 8.4% having been classified under the “steadily high” trajectory. This may suggest that these patients encountered particular stressors or otherwise had difficulties adapting to their lives without success. However, more research will be needed to test this possibility, and healthcare providers should identify patients with higher baseline CES-D scores in an effort to better clarify factors associated with worse depressive symptoms to guide early intervention efforts.

This study found that older patients were more likely to experience a poorer depressive symptom trajectory. This may be attributable to the potential for older individuals to exhibit worse physical function and dyspnea status as compared to younger COPD patients [24]. Moreover, older individuals may exhibit more comorbidities. We found that the number of comorbid conditions at baseline was an important predictor of depressive symptom trajectories in COPD patients. These findings was similar to the results of prior study suggesting that chronic illnesses was predictive of depressive symptoms in individuals with COPD [25]. Chronic diseases can complicate activities of daily living, to be specific, the chronic diseases may contribute to disability among COPD patients, which was associated with social exclusion, social isolation, lack of social participation, and loneliness, these have been shown to strong correlates of depressive symptoms [25]. Moreover, COPD patients with greater numbers of comorbidities may worry more regarding their health, potentially worsening their depressive symptoms. Thus preventing and controlling chronic diseases is important in improving mental health of COPD subjects.

Nightly sleep duration was identified as an important determinant of depressive symptom trajectory, with participants exhibiting a longer night-time sleep duration tending to exhibit poorer trajectory. The mechanistic basis for this link remains to be clarified. Prolonged sleep duration has been reported to correlate with low levels of physical activity among COPD patients [26]. High levels of physical activity has been reported to lower the risk of depression by enhancing brain aminergic synaptic transmission, increasing serotonin and dopamine layers, augmenting endorphin secretion, providing distractions from stressful stimuli, or bolstering self-esteem and self-efficacy [27]. Therefore, the COPD patients that sleep for longer periods of time devote less time to physical activity, so they are more likely to exhibit more depressive symptoms. Moreover, for COPD patients, long sleep duration may be a possible compensatory phenomenon of a failing sleep-regulatory function [26]. Thus, prolonged sleep duration was similar to the decreased neuromuscular function, i.e., a marker of failing physiological functions, which may contribute to the depressive symptoms. This finding indicated that health care providers should carefully focus on the possible effect of sleep duration on depressive symptoms among COPD patients.

Moreover, patients with a high BMI were more likely to be classified under the “consistently moderate” depressive symptom trajectory. Being overweight or obese is known to be linked with worse mental health outcomes as compared to those in matched normal-weight populations [28]. A BMI > 25 has been found to be independently related to the development of depressive symptoms among individuals with COPD [29]. Behavioral findings of individuals with high BMI such as reductions in physical activity and a sedentary lifestyle may contribute to depressive symptom development in COPD populations [30]. Moreover, higher BMI was found to be related to an increased risk of chronic illnesses [31], including heart disorders, hypertension, diabetes, and stroke, these makes daily activities effortful, having the potential to impact the development of depressive symptoms in COPD individuals. This result emphasizes the importance of monitoring mood status among COPD patients with a high BMI in healthcare contexts, and providers should thus take the relationship between BMI and depressive symptom trajectories into account in clinical settings.

We found that higher education levels were associated with poorer depressive symptom trajectories among the studied patient population. However, this finding is inconsistent with prior reports that poorer education is predictive of worse depressive scores [32]. One potential explanation for this result may be that more educated individuals are more likely to exhibit higher expectations and demands regarding life such that they may be unsatisfied with their current life, resulting in or exacerbating depressive symptoms. Further research will be essential to test this possibility. We additionally detected an association between low HGS and more intense depressive symptoms among COPD individuals. Low HGS was an indicator of poor muscle strength, which was found to have a strong correlation with depression in prior COPD literature [33]. Moreover, lower HGS was indicative of poorer daily activities in COPD patients [26], and has been linked to decreased exercise performance, fatigue, decreased social activities, and feelings of helplessness, all of which contribute to the development of depressive symptoms [34]. Given that depression is often under-diagnosed in COPD patient populations, lower HGS may offer value as an indicator that can incentivize physicians to conduct depressive symptom screening in this at-risk patient population [35].

### Limitations

There are several limitations to this study. Firstly, depressive symptoms were measured using a self-reported scoring instrument and may thus be subject to recall bias. Then, disease severity has the potential to impact depressive symptoms. Moreover, the diagnosis of COPD was self-reported in CHARLS survey, not based on spirometry. Furthermore, Given that the CHARLS survey did not contain any tools for the grading of COPD severity such as the Global Initiative for Chronic Obstructive Lung Disease criteria, thus constraining our ability to examine the link between depressive symptoms and disease severity. Additionally, although CHARLS database assessed whether patients had psychiatric problems, it does not specify what it was, which limited our ability to explore the influence of specific diseases on the trajectories of depressive symptoms. Lastly, we only assessed subjects 45 years of age or older, limiting the generalizability of these findings.

## Conclusions

In summary, we identified the “steadily high”, “consistently moderate”, and “consistently low” depressive symptom trajectories among self-reported COPD patients. These results offer a framework within which health-care workers can gain insight regarding depressive symptom trajectories over 4-year periods in COPD individuals to guide treatment efforts. Furthermore, we found the determinants of depressive symptom trajectories in self-reported COPD patients. To prevent the progressive worsening of patient depressive symptoms, it is important that they be regularly evaluated to guide appropriate interventional efforts in clinical contexts.

## Data Availability

The datasets analyzed during the current study are available in the CHARLS repository, http://charls.pku.edu.cn.
